# Medical image registration utilizing tissue P systems

**DOI:** 10.3389/fphar.2022.949872

**Published:** 2022-08-05

**Authors:** Saleem Sanatan Kujur, Sudip Kumar Sahana

**Affiliations:** Department of Computer Science and Engineering, Birla Institute of Technology Mesra, Ranchi, India

**Keywords:** P systems, MRI, TPS, optimization, medical image registration

## Abstract

The tissue P system (TPS) possesses intrinsic attributes of parallel execution in comprehensive data and instruction space, which provides fast convergence during the transition from local to global optima. Method- In this study, we have proposed and built a TPSysIR framework using the TPS for image registration that optimizes upon the mutual information (MI) similarity metric to find a global solution. Result- The model was tested on single- and multimodal brain MRI scans and other prominent optimization-based image registration techniques. Conclusion- Results show that, among all methods, TPSysIR provides better MI values with minimum deviation in a range of experiment setups conducted iteratively.

## Introduction

Medical image registration involves processing image data from multiple sources, each having a different coordinate system. These sources often have different sensors and viewpoints, transforming the data collected into a single spatial coordinate system. Image registration requires optimized parameter values for the required transformation, translation, or rotation to be applied over the source images with respect to the reference image to achieve matching. Image registration has been utilized in many recent advances in image reconstruction (Prakash et al., 2019), land cover mapping (Wang et al., 2020), and weather prediction (Kakimoto et al., 2019). A large number of image registration methods are already available, which can be classified as single or multimodal, automatic vs. inter-active, spatial domain vs. frequency domain-based, intensity vs. feature-based, and transform-based. Image registration finds essential applicability in the areas of remote sensing and medical image processing. Image registration can be viewed as an optimization problem (Song et al., 2017) whose aim is to maximize the similarity or minimize the cost in the process. It takes single or multiple image data and transforms them according to the parameters to maximize the similarity to the target reference image. Various parameters which can be optimized are correlation ratio (Gong et al., 2019), mutual information (Ramamoorthy et al., 2010), energy of joint probability distribution (Susskind et al., 2011), normalized correlation (Lewis, 2001), and normalized mutual information (Knops et al., 2005).

Optimization methods such as Powell’s were among the earliest attempts to solve image registration problems. However, the algorithm provided local optimum results, and the objective search speed was also low. These shortcomings lead to the utilization of nature-based optimization techniques for image registration as the next-generation solution. The genetic algorithm (GA)-based method has been proposed by Rouet, Jacq, and Roux (Rouet et al., 2000). Utilizing the local optima, LI Zuo-zhu (Zuo-zhu, 2007) applied GA on mutual information (MI) metric optimization to achieve an image registration solution. Rajapakse and Guojun (Rajapakse and Guojun, 1999) performed image registration by utilizing GA on time-series images. Still, the nonexistence of fine-tuning ability coupled with considerable execution time led researchers to explore better methods. Chen, Lin, and Mimori (Lin et al., 2012) utilized particle swarm optimization (PSO) on the image dataset, optimizing the MI measure. Wachowiak et al. (Wachowiak et al., 2004), Chel, and Nandi (Chel and Nandi, 2013) used hybrid PSO on 3-D medical images; similarly optimization was performed on normalized MI decreasing the overall execution time. Basset et al. (Abdel-Basset et al., 2017) utilized modified MI metric and PSO for image registration. This method fell into local maxima with increasing degrees of rotation. Zhang et al. (Zhang et al., 2010) integrated PSO with Powell to overcome these shortcomings and applied them to image registration.

In recent years, machine learning (Zhu et al., 2022) and deep learning (Zhu et al., 2021) have found applications in image processing and registration. Balakrishna et al. (Balakrishnan et al., 2019) used a convolutional neural network for 3D image registration. Ali and Rittscher (Ali and Rittscher, 2019) utilized concatenated convolutional layers for deformable image registration. Mansilla, Milone, and Ferrante (Mansilla et al., 2020) proposed the AC-RegNet architecture to achieve image registration.

Membrane computing (MC) (Paun, 2000) was introduced by Gheorghe Paun, inspired by the computational mechanisms of living cells or tissue systems. Biological and computational processes at the cellular and tissue level are performed in a maximally parallel and randomly distributed manner. These random processes and communications are triggered when appropriate compounds and catalyst inhibitors are present in the cellular environment. Membrane computing forms the computational model called P systems; these have been efficiently utilized to obtain solutions to many NP-complete (Paun, 2001) problems by creating a trade-off between time and space complexity. P systems are built upon low-level biological interactions or processes by equipping them to capture the computational essence of complex cell metabolism and information interchange. The P system may use any one of the following mechanism or mechanisms to create variants of the system: selective object recognition, controlled exchange of particles through protein channels, cytoplasmic metabolism or division, and dissolution of membranes. The P system has been proved computationally complete and is utilized to solve many optimization-based and NP-complete problems (Paun, 2001), such as subset sum (Jiménez and Núñez, 2005), TSP, and tricolor problems. Computation in a P system proceeds in a maximally parallel and non-deterministic path, which can be tuned according to the execution model of the problem.

### Membrane system

**FIGURE 1:**
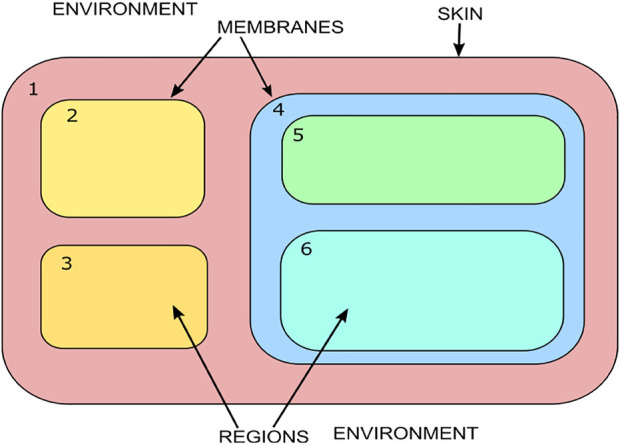
Membrane system.

**FIGURE 2:**
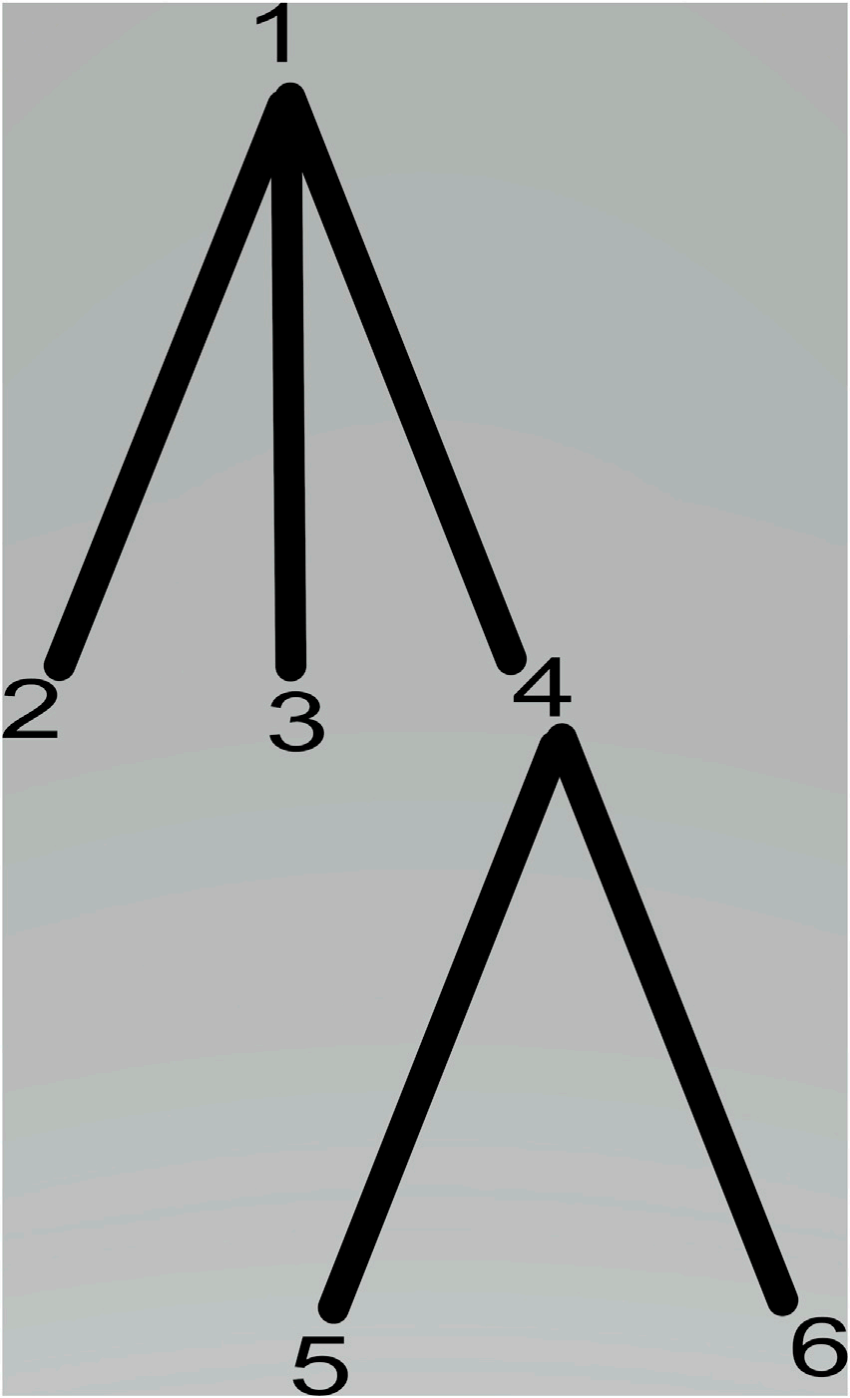
Membrane hierarchy.

The membrane system shown in Figure 1 can be viewed as a hierarchically organized set of membranes existing inside an outer space called the environment. The tree in Figure 2 can represent the hierarchical organization of a membrane system in Figure 1. The tree’s root is associated with the skin membrane, and the leaves are associated with the elementary membranes. The membranes at the same level can float around in the same membrane compartment. The hierarchical string expression 1) is written for Figure 2 membrane structure:

$${\left[\;\right.}_{1}{\left[\;\right.}_{2}{\left.\;\right]}_{2}{\left[\;\right.}_{3}{\left.\;\right]}_{3}{\left[\;\right.}_{4}{\left[\;\right.}_{5}{\left.\;\right]}_{5}{\left[\;\right.}_{6}{\left.\;\right]}_{6}{\left.\;\right]}_{4}{\left.\;\right]}_{1}.$$ (1)

### P systems

The operations of a P System can be visualized as an extended distributed computation machine that presents a range of solutions to a particular problem. The nature of the multiset solution present in the output environment or membrane varies, based on the halting condition associated with the problem. The distribution and transition of multisets in the membrane regions determine the generated languages and their related grammar.Formally a P system *∏* can be defined as

$$\prod =\left(O,M,m_{1},m_{2},\ldots m_{n},R_{1},R_{2},\ldots ,R_{n},\delta_{0}\right).$$ (2)

Here, O is the finite set of objects. M is the set of membranes. *m*_*i*_ is the multiset of objects in the membrane. *R*_*i*_ is the rule inside the corresponding membrane. *δ*_0_ is the set of output membrane.

**FIGURE 3:**
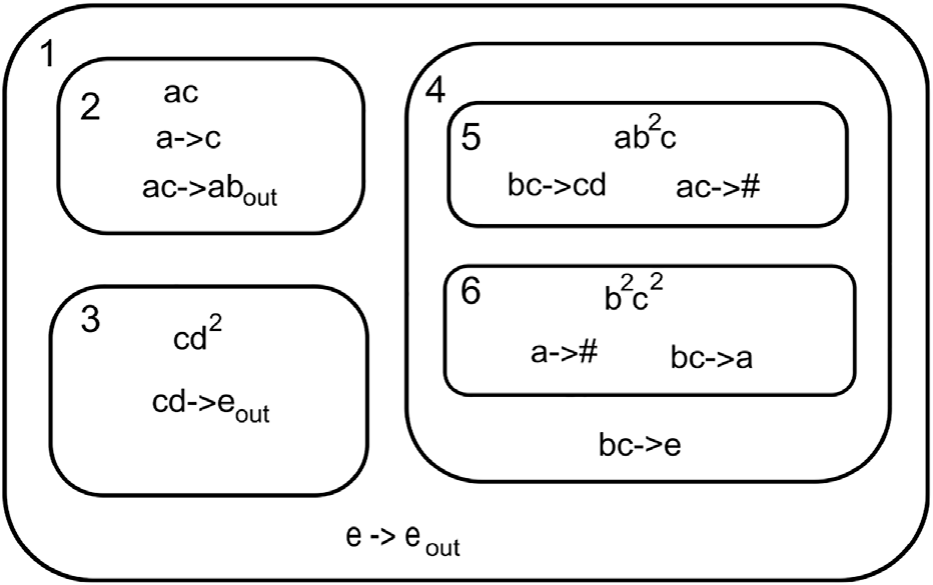
P system.

A P System can be viewed as a hierarchical system comprising of a three-dimensional space referred environment containing membranes. The membranes contain a set of objects called multiset, coexisting with rules and other membranes.Figure 3 shows a P System.

The P System can be formally written as

$$\begin{array}{c} \prod =\left(\left\{a,b,c,d,,e\right\},\left\{1,2,3,4,5,6\right\},\left\{,ac,cd^{2},ab^{2}c,b^{2}c^{2}\right\}\right.\\ \left\{\left\{e\to e_{\mathrm{out}},\right\}\left\{ac\to ab,a\to b_{\mathrm{out}}\right\},\left\{cd\to e_{\mathrm{out}}\right\},\right.\\ \left\{bc\to e_{\mathrm{out}}\right\},\left\{bc\to cd,ac\to \#\right\},\left\{bc\to e_{\mathrm{out}}\right\},\\ \left.\left.\left\{bc\to a,a\to \#\right\}\right\},\left\{1\right\}\right) \end{array}.$$ (3)

Here, O = {*a*, *b*, *c*, *d*, *e*}, M = {1, 2, 3, 4, 5, 6}, and *m*_*i*_ = $\left\{,ac,cd^{2},ab^{2}c,b^{2}c^{2}\right\}\;\begin{array}{ccc} R_{i} & = & \left\{\left\{e\to e_{\mathrm{out}},\right\},\left\{ac\to ab,a\to b_{\mathrm{out}}\right\},\right.\\ & & \left\{cd\to e_{\mathrm{out}}\right\},\left\{bc\to e_{\mathrm{out}}\right\},\left\{\;\right.bc\to cd,\\ & & ac\to \#\left.\;\right\},\left.\left\{bc\to a,a\to \#\right\}\right\} \end{array}\;\delta_{0=\left\{1\right\}}$ .The rules in the membranes are represented as *x* → *y* or *x* → *y#*, with *x ϵ O* ^+^ and *v ϵ* (*O* × *Tar*)*, where *Tar* = {*here*; *in*; *out*}. Many forms of multiset rewriting and communicating rules have been utilized to convey more information about the state. There are mainly two types of rules: evolution and communication rules. The evolution rules govern the evolution of the membrane state, and communication rules facilitate communication, i.e., data transportation from one membrane to another. Evolution rules are of the form *l* → *m* or *l* → *#*; here, the occurrence of the *#* symbol leads to the dissolution of the membrane wherever the rule is executed, and all multisets currently existing inside it are passed onto its parent membrane. Communication rules are of form *w* → *x*_*out*_ *y*_*in*_; here the multiset *w* forms two multisets, *x* and *y* ; here, *x* moves outside the membrane to the parent whereas *y* moves to the child existing inside the current membrane.

### Computation process in a P system

Computation in a P system is performed in a non-deterministic and maximally parallel manner. The configuration changes from an initial state to the next state in an asynchronous manner, referred to as the transition of a P system. The computation is thus the continuous transition of the P system by applying the rules in a non-deterministic and maximally parallel manner until the system halts. The halting condition is achieved when no further rules can be applied and the output is obtained as the contents of the output membrane. A non-deterministic manner ensures that the rules are chosen at random. This randomness may lead to different transition paths. The order of application of rules is also an important aspect. Maximally parallel application of rules ensures that all possible rules are executed simultaneously in every transition step of the computation. The rules rewrite the multiset content inside a membrane. The execution of *u* → *v* rules is dependent mainly on the availability of the multiset composing the left side of the membrane *u*, which transforms into the right side multiset *v*.

**FIGURE 4:**
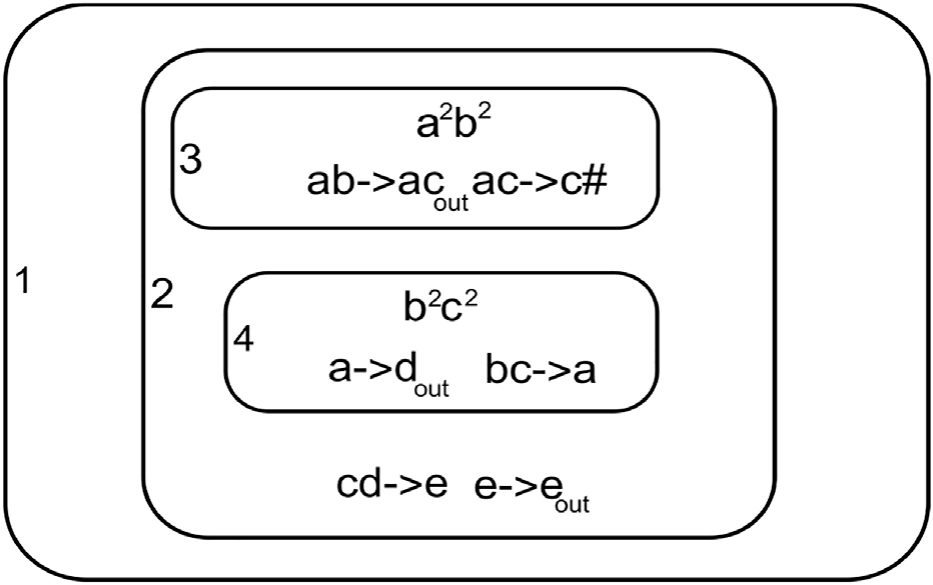
P system.

Figure 4 shows a simple P System with four membranes 1, 2, 3, and 4. Membrane one is the outermost membrane that holds the output on halting. The system can transit through multiple process paths due to the non-deterministic nature of the computation.

### Example steps of computation

• Step 1: Observing the initial configuration in membranes three and four Figure 4

*m*_3_, *R*_3_ = {*a* ^2^ *b* ^2^}, {*ab* → *ac*, *ac* → *c#*} *ab* is assigned to rule *ab* → *ac m*_4_, *R*_4_ = {*b* ^2^ *c* ^2^}, {*bc* → *a*, *a* → *d*_*out*_} *bc* is assigned to rule *bc* → *a*.

• Step 2: After transition in step 1 we have

*m*_3_, *R*_3_ = {*a* ^2^ *c* ^2^}, {*ac* → *c#*} *ac* is assigned to rule *ac* → *c# #* dissolves membrane three and multiset *ac* ^2^ moves into membrane two

*m*_4_, *R*_4_ = {*a* ^2^}, {*a* → *d*_*out*_} *a* is assigned to rule *a* → *d*_*out*_ *d* ^2^ is passed out of membrane four and no more transitions can happen in it.

• Step 3: After step 2 we have the membrane possible for transition

*m*_2_, *R*_2_ = {*ac* ^2^ *d* ^2^}, {*cd* → *e*, *e* → *e*_*out*_} *cd* is assigned to rule *cd* → *e*.

• Step 4:After step 3 membrane two new state

*m*_2_, *R*_2_ = {*ae* ^2^}, {*e* → *e*_*out*_} *e* is assigned by *e* → *e*_*out*_ *e* ^2^ is passed out to membrane one.

• membrane one is the output membrane so the computation halts here

*m*_1_, *R*_1_ = {*e* ^2^}

The computation halts since no more rules can be executed.

### Mutual Information

Mutual information measures the statistical dependency between two sets of data (here the image data sets) independent of the intensity values of images. The MI value between two images or voxels is maximum when the geometrical alignment between them is good. MI measures two sets of image data , A and B, obtained as follows:

$$MI\left(A,B\right)=\underset{a,b}{\sum }\mathrm{Prob}\left(a,b\right)\log\frac{\mathrm{Prob}\left(a,b\right)}{\mathrm{Prob}\left(a\right)\mathrm{Prob}\left(b\right)}.$$ (4)

Here, Prob (*a*, *b*) is the joint probability of *a ϵ A* and *b ϵ B*. Prob(*a*) and Prob(*b*) are the independent probabilities.

## Methods

### Tissue P system

Tissue P system (Pan and Perez-Jim´enez, 2010) can be viewed as the graph of P system membranes connected with bidirectional protein channels (Freund et al., 2005). The protein channels facilitate the communication (transportation) of multisets of objects between the membranes. The communication can be performed in a replicative manner where a copy of the multiset can be sent to all adjacent membranes attached to the communication channel (protein channel) or in a non-replicative manner where only one copy of the membrane is communicated to a particular membrane. Mathematically, the tissue P system (Bernardini and Gheorghe, 2005) of degree (number of membranes) n can be defined as

$$\Gamma =\left(O,\mu_{1},\mu_{2},\ldots ,\mu_{n},comm,\mu_{\mathrm{out}}\right).$$ (5)

Here,

1. *O* is a finite set of objects (alphabets)
2. *comm* ⊆{1, 2, 3, .., *n*}×{1, 2, 3, … , *n*}
3. *μ*_*out*_ = {*μ*_1_, *μ*_2_, … , *μ*_*n*_} is the output membrane
4. {*μ*_1_, *μ*_2_, … , *μ*_*n*_} are the membranes of form *μ*_1_ = {*s*_*i*,0_, *mul*_*i*,0_, *Rule*_*i*_}
  (a) *s*_*i*,0_ is initial state of *i* ^*th*^ membrane
  (b) *mul*_*i*,0_ multiset of *i* ^*th*^ membrane
  (c) *Rule*_*i*_ set of rules in *i* ^*th*^ membrane

**FIGURE 5:**
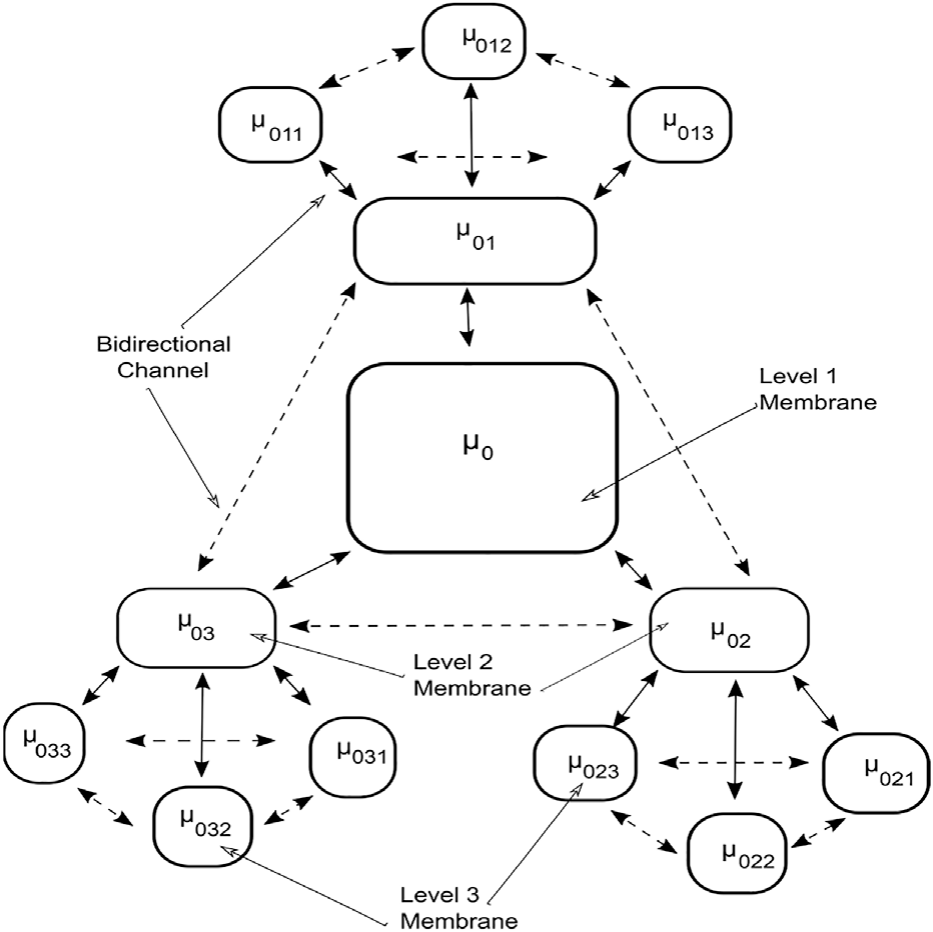
Tissue P system for TPSysIR algorithm.

The tissue P system in Figure 5 is organized in the form of a multilevel membrane structure, with levels one and two having three membranes as child membranes. The output membrane or level 1 membrane is labeled using*μ*_0_. It contains three child membranes labeled by *μ*_01_, *μ*_02_, and *μ*_03_ respectively; these form level two in the system. The level two membranes further contain three child membranes each. Child membranes are labelled as *μ*_*ab*_, where *a* is the parent node and *b* is the child membrane. The system is interconnected with a bidirectional transportation channel that facilitates the transportation or communication of objects between the membranes. Each membrane contains multisets of objects along with the rules governing the evolution and communication of objects. The algorithm searches for the optimal solution among the floating image objects configured with the transform parameters inside the membranes. The objects in the solution space continuously evolve by utilizing the rules and are examined for the existence of a better optimized solution.

### The objects

The object in the solution space can be represented as the set of transformation parameters:

$$Ojb_{rs}=x_{rs},y_{rs},\theta_{rs},Z_{rs}.$$ (6)

1. r = 1 … n and s = 1 … m
2. n is the membrane count of tissue P system
3. m is the object count in membrane r
4. *θ*_*rs*_ is the degree of rotation
5. *Z*_*rs*_ is the image scaling factor
6. *x*_*rs*_ is the displacement in *x* axis
7. *y*_*rs*_ is the displacement in *y* axis

The algorithm utilizes the MI metric as the optimization function to measure and maximize the similarity of the parameters in the evolved objects. The object evolves inside the membranes, and the local best is selected for each level 3 membrane. This local best value is communicated to the neighboring membrane and level 2 membranes through the bidirectional channel. The level 2 membranes communicate between themselves and their children to search for the global best solution. This solution is then transferred to the level 1 output membrane, thus representing the final solution.

### The evolution rules

The evolution rules govern the evolution of float image object configurations inside the membrane; the evolution is performed so as to achieve the optimal best object according to the optimization criteria. Each level 3 membrane contains three optimal objects; $Obj_{ab}^{t,best}$ , the local optimal best object obtained at the *t* ^*th*^ moment inside the *ab* ^*th*^ membrane, $Obj_{ab,n}^{t,best}$ , one of the optimal best object randomly selected among all the optimal objects received from the neighbors to the *ab* ^*th*^ membrane, and $Obj_{ab,u}^{t,best}$ , the optimal best object transferred from the parent level 2 membrane *u* to *ab* ^*th*^ child membrane.

The PSO (particle swarm optimization) technique is utilized here to govern the formation and execution of *rule*_1_ and *rule*_2_, and its velocity position equation is modified to define the evolution rules. These rules evolve the objects according to the position (configuration) and time.

$$\begin{array}{ll} v_{k,ab}^{t+1} & =\omega^{t}v_{k,ab}^{t}+l_{0}z_{0}\left(Obj_{ab}^{t,best}-Obj_{k,ab}^{t}\right)+l_{1}z_{1}\left(Obj_{ab,n}^{t,best}-Obj_{k,ab}^{t}\right)\\ & \;+l_{2}z_{2}\left(Obj_{ab,u}^{t,best}-Obj_{k,ab}^{t}\right), \end{array}$$ (7)

$$Obj_{k,ab}^{t+1}=Obj_{k,ab}^{t}+v_{k,ab}^{t+1},$$ (8)

$$rule_{1}\equiv {\left[Obj_{k,ab}^{t}\right]}_{ab}^{t}↔{\left[Obj_{k,ab}^{t+1}\right]}_{ab}^{t+1},$$ (9)

$$\begin{array}{cc} Obj_{ab}^{t+2,best}= & \max\left\{\left\{MI\left(Obj_{k,ab}^{t+1}\right)\right\}\cup MI\left(Obj_{ab}^{t,best}\right)\right\} \end{array},$$ (10)

$$rule_{2}\equiv {\left[Obj_{ab}^{t,best}\right]}_{ab}^{t+1}↔{\left[Obj_{ab}^{t+2,best}\right]}_{ab}^{t+2}.$$ (11)

The velocity component is updated using (7) *ω* ^*t*^ is the weight balancing factor which gradually decreases; *l*_0_,*l*_1_,and *l*_2_ are the learning factor; *z*_0_, *z*_1_, and *z*_2_ are the random numbers between 0 and 1. $Obj_{k,ab}^{t}$ is the *k* ^*th*^ float object in *ab* ^*th*^ membrane having a configuration of the floating image. This is updated by *rule*_1_ in Eqs. 8, 9 .This updates the new configuration of each object inside the membrane. The local best object of the membrane is selected by utilizing Eq 10, and the old one is replaced by the new local best utilizing *rule*_2_ at Eq 11.

### The communication rules

The communication (Paun and Paun, 2002) rules facilitate the transportation of objects between the membranes at inter- or intra-level utilizing the bidirectional channel connecting them.

### Intra-level object communication rule

The local optimal object $Obj_{ab}^{t,best}$ is updated during the evolution stage inside each membrane at level 3. These objects are further communicated to every neighboring membrane under the shared parent membrane. This process involves the creation of a duplicate copy of the local optimal object $Obj_{ab}^{t,best}$ in each membrane, exchanging it with every other membrane under the common parent. The intracommunication rule is described as follows:

$$rule_{3}\equiv {\left[Obj_{aj}^{t,best}\right]}_{ai}^{t}↔{\left[Obj_{ai}^{t,best}\right]}_{aj}^{t}.$$ (12)

In Eq 12 *rule*_3_ the objects $Obj_{aj}^{t,best}$ and $Obj_{ai}^{t,best}$ are the local optimal bests in membranes *aj* and *ai*, respectively. Both are located at level 3 under the same parent membrane. The objects are exchanged and *rule*_3_ is executed in Eq 12.

### Inter-level object communication rule

The copy of the local optimal object $Obj_{ab}^{t,best}$ is updated during the evolution stage in each membrane at level 3, and this object is also communicated to the parent membrane at level 2. All the membranes in the child level 3 receive a copy of $Obj_{u}^{t,best}$ optimal object updated at the parent level. This process involves simultaneous duplication and communication between a membrane at the parent and another at the child level. The intercommunication rule is described as

$$rule_{4}\equiv {\left[Obj_{u}^{t,best}\right]}_{ab}^{t}↔{\left[Obj_{ab}^{t,best}\right]}_{u}^{t},$$ (13)

$$rule_{5}\equiv {\left[Obj_{u}^{t,best}\right]}_{u}^{t}↔{\left[Obj_{u}^{t,best}\right]}_{0}^{t}.$$ (14)

In (13), *rule*_4_ exchanges the objects $Obj_{ab}^{t,best}$ and $Obj_{u}^{t,best}$ from membrane *ab* in level 3 and membrane *u* at level 2, respectively. The objects are first copied and are then exchanged between each child and parent membrane, executing *rule*_4_ for each child’s membrane. The membrane at level 2 will have local optimal best objects from all its child membranes, and all its children will have a copy of the optimal object from its parent.

The (14) *rule*_5_ communicates the object $Obj_{u}^{t,best}$ optimal best of membrane *u* at level 2 is copied and sent to output membrane at level 1 as the global best.

### The selection and substitution rules

After the inter level object communication stage, the membrane at level 2 has *n* copies of the object from each of its child nodes. Level 2 membrane selects the best among all the objects received from the child membranes and compares it with its local optimal best. The MI metric is utilized to perform the selection between the two best optimal values. The maximal object obtained from the above process is substituted as the current local best of this membrane. The rule can be described as

$$\begin{array}{cc} Obj_{ab}^{t+2,best}= & \max\left\{\left\{MI\left(Obj_{k,ab}^{t+1}\right)\right\}\cup MI\left(Obj_{ab}^{t,best}\right)\right\} \end{array},$$ (15)

$$rule_{6}\equiv {\left[Obj_{u}^{t,best}\right]}_{u}^{t}\to {\left[Obj_{u}^{t+1,best}\right]}_{u}^{t+1}.$$ (16)

Eq. 15 examines the MI of all objects $Obj_{k,ab}^{t+1}$ received from the child membranes along with the MI of the current best object $Obj_{u}^{t,best}$ of membrane *u* for the maximum among all of them. This value is replaced as the new local best of membrane *u* by *rule*_6_ in Eq 16.

The designed TPS utilizes the search capability of the PSO algorithm and explores the search space filled with floating image object solutions. The local object evolution is performed at the third level of the system; this generates local optimum objects. The optimized objects then move to the neighboring membrane and higher level 2 membranes.

### The halting condition

The system is executed in the manner of steps; it is halted after the desired number of steps are performed. The optimal object obtained at the output level 1 membrane at that instance is recorded as the best solution to the problem.

### The TPSysIR algorithm

**FIGURE 6:**
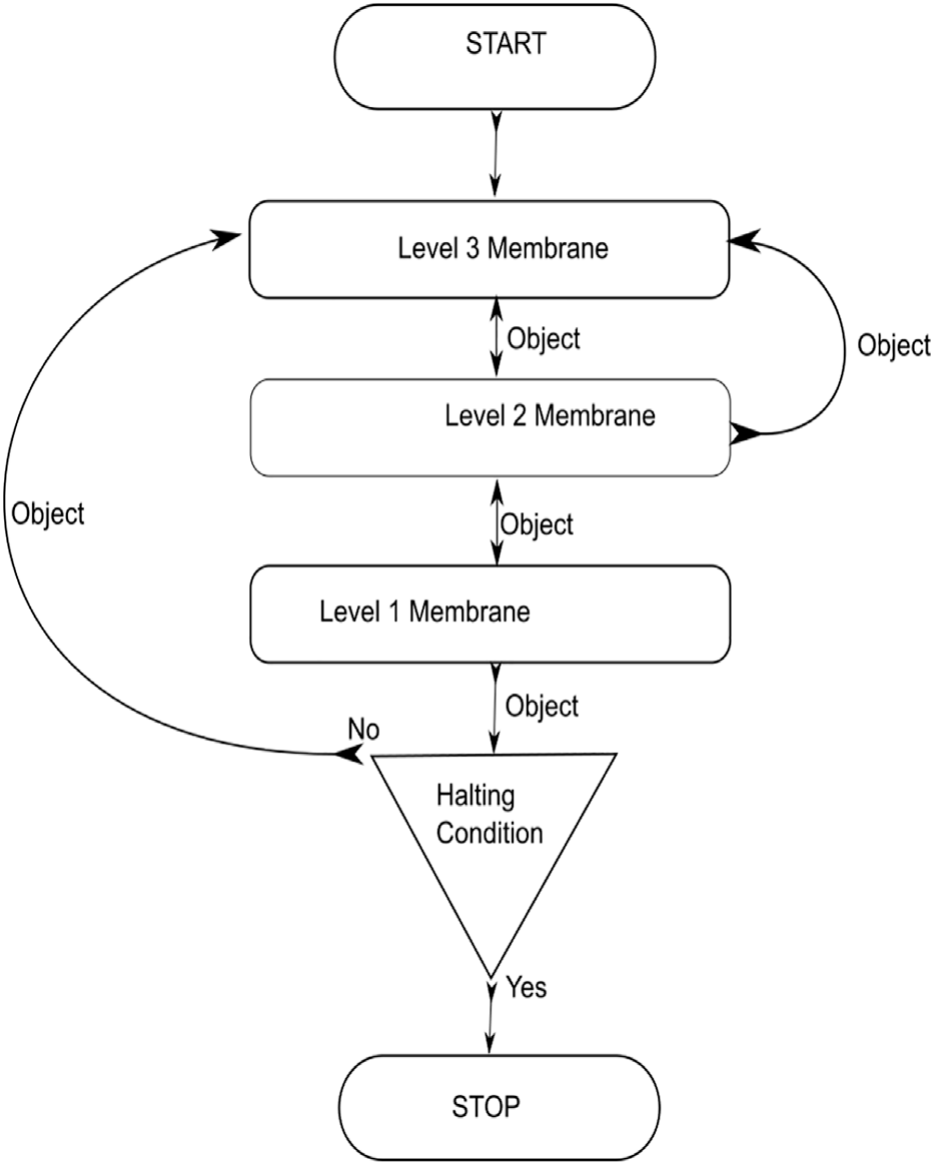
TPSysIR algorithm.

The algorithm is designed using the TPS framework in the form of three membrane levels; the algorithm is shown in Figure 6. The system utilizes each level for specific evolution and optimization objectives. The level 3 membranes are utilized to evolve the floating image objects to achieve local optimal values at their level inside each membrane. Communication of local optimal objects among membranes under a common parent is done to optimize the configuration object further. This optimal object, obtained from each child membrane at level 3, is communicated to the level 2 membranes to form the global optimal solution. Level 2 membranes examine the optimality of the received objects from the child membranes to create a global maximal. The optimal value of level 2 is sent to the level 1 output membrane. The copy of the optimal value of the level 2 membrane is also sent back to the children’s membrane. The entire process, from the evolution of objects in the level 3 membrane to the transfer of global optima from level 2 to level 1, constitutes a step. The specified number of steps must be completed before the system halts; otherwise, the system restarts the evolution process utilizing the previously obtained locally optimized solutions.

## Experimental setup

All the experiments were conducted using python (Python Programming Language, 2021) and plingua (P Lingua Programming Language, 2021) on a platform with Intel i5 four-core CPU at 2.5 GHz each and 8 gigabytes of RAM. The image data consisting of standard brain atlas was obtained from the Montreal Neurological Institute (MNI) (McConnell Brain Imaging Centre, 2021). The data was utilized by different optimization-based methods, utilizing MI as an optimization metric for image registration. The experiments are divided into two sets; first set of two experiments utilizes the single modal data and second set of three experiment utilizes the multimodal data.

### Experiments with a single-modal image

**FIGURE 7:**
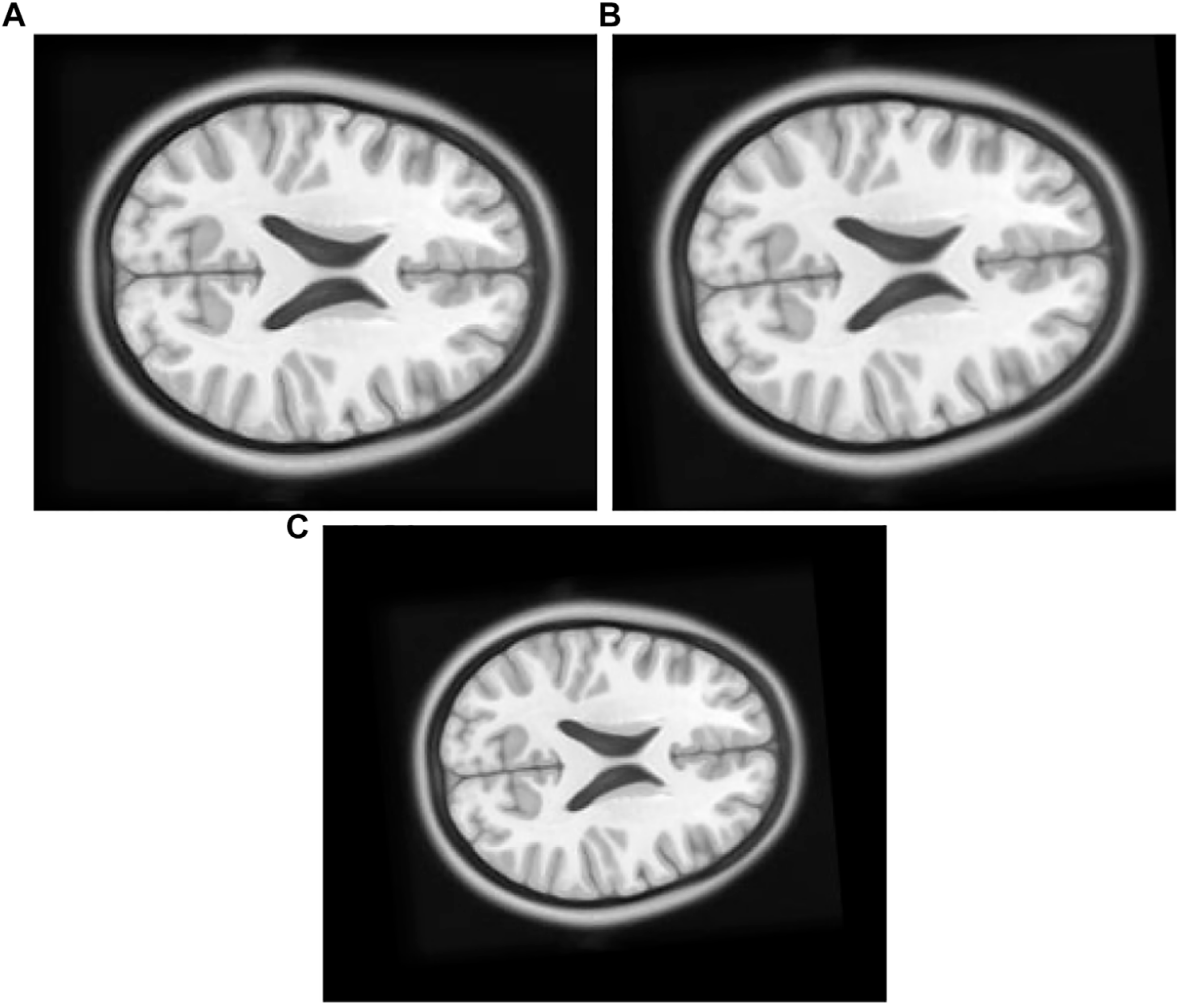
**(A)** Original Image: row first left. **(B)** Float Image Unscaled: row first right. **(C)** Float Image Scaled: second row center.

**FIGURE 8:**
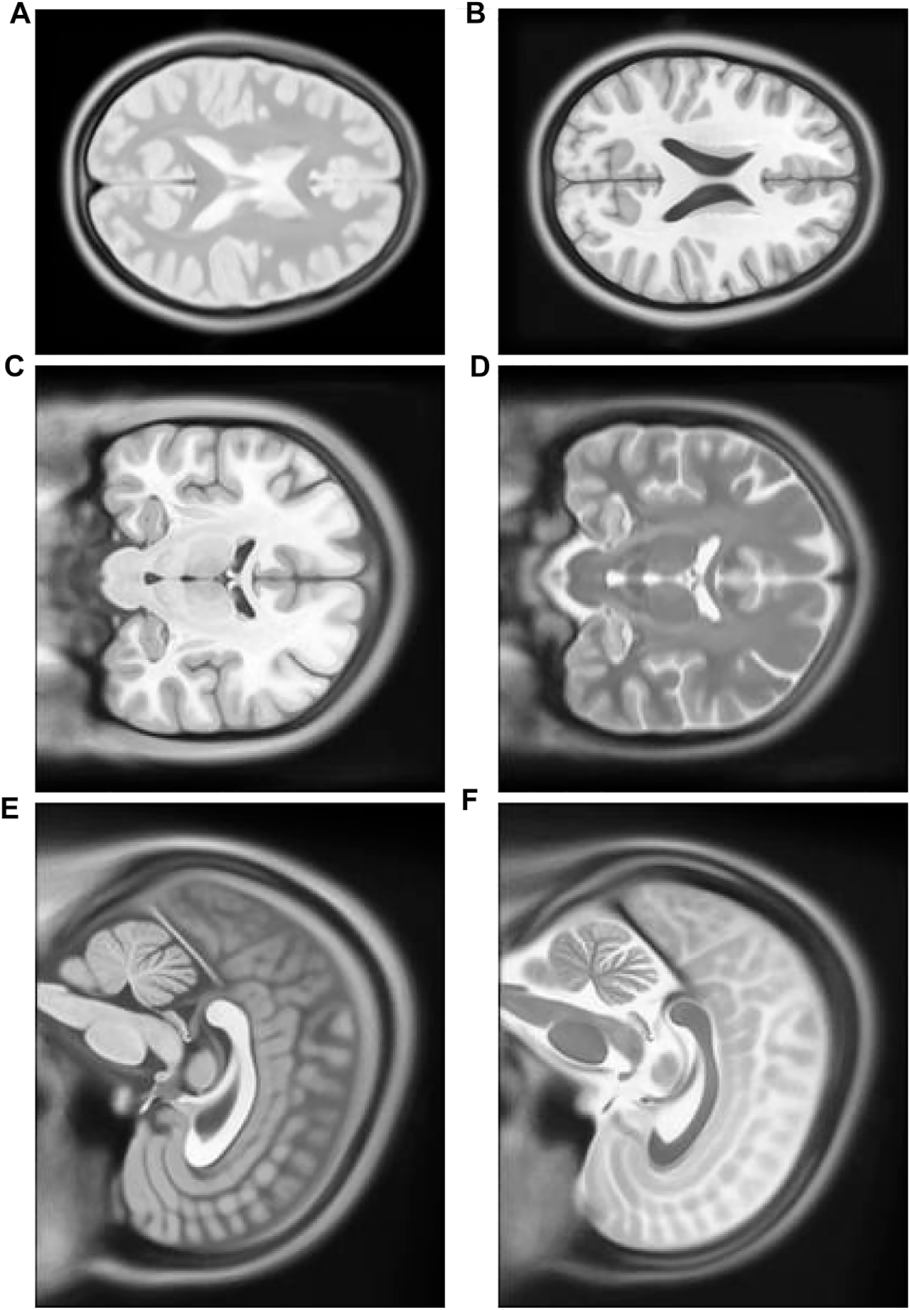
**(A)** Multimodal Experiment 1 T2: row first left. **(B)** Multimodal Experiment 1 T1: row first right. **(C)** Multimodal Experiment 2 T1: row second left. **(D)** Multimodal Experiment 1 T2: row second right. **(E)** Multimodal Experiment 3 T1: row third left. **(F)** Multimodal Experiment 1 T2: row third right.

1) Experiment 1: The float Figure 7B in this experiment is created by moving the original Figures 7A, 8 pixel units in *x*-axis up direction, six pixel units in *y*-axis in the left direction, and rotated 5° in a counterclockwise direction.

2) Experiment 2: The original Figure 7A in this experiment is moved eight pixel-units in *x*-axis up direction, six pixel-units in *y*-axis, rotated 5° in a counterclockwise direction, and scaled into 0.8 times of its original size to create float Figure 7C.

### Experiment with multimodal images

**FIGURE 9:**
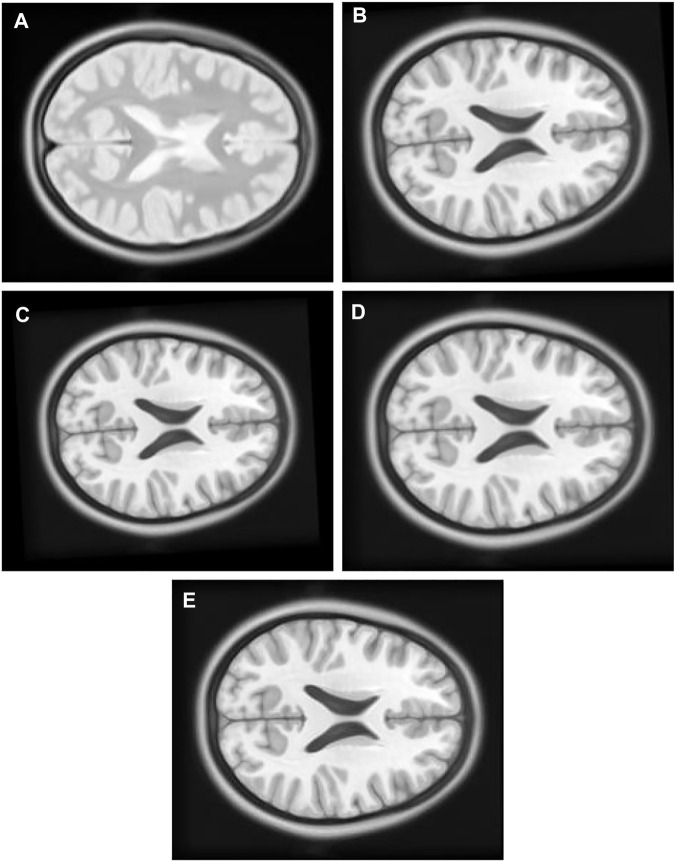
**(A)** Multi Modal Experiment 1 Float: row first left. **(B)** Multi Modal Experiment 1 GA output: row first right. **(C)** Multi Modal Experiment 1 PSO output: row second left. **(D)** Multi Modal Experiment 1 PSO and Powell Output: row second right. **(E)** Multi Modal Experiment 1 TPSysIR Output: row third center.

**FIGURE 10:**
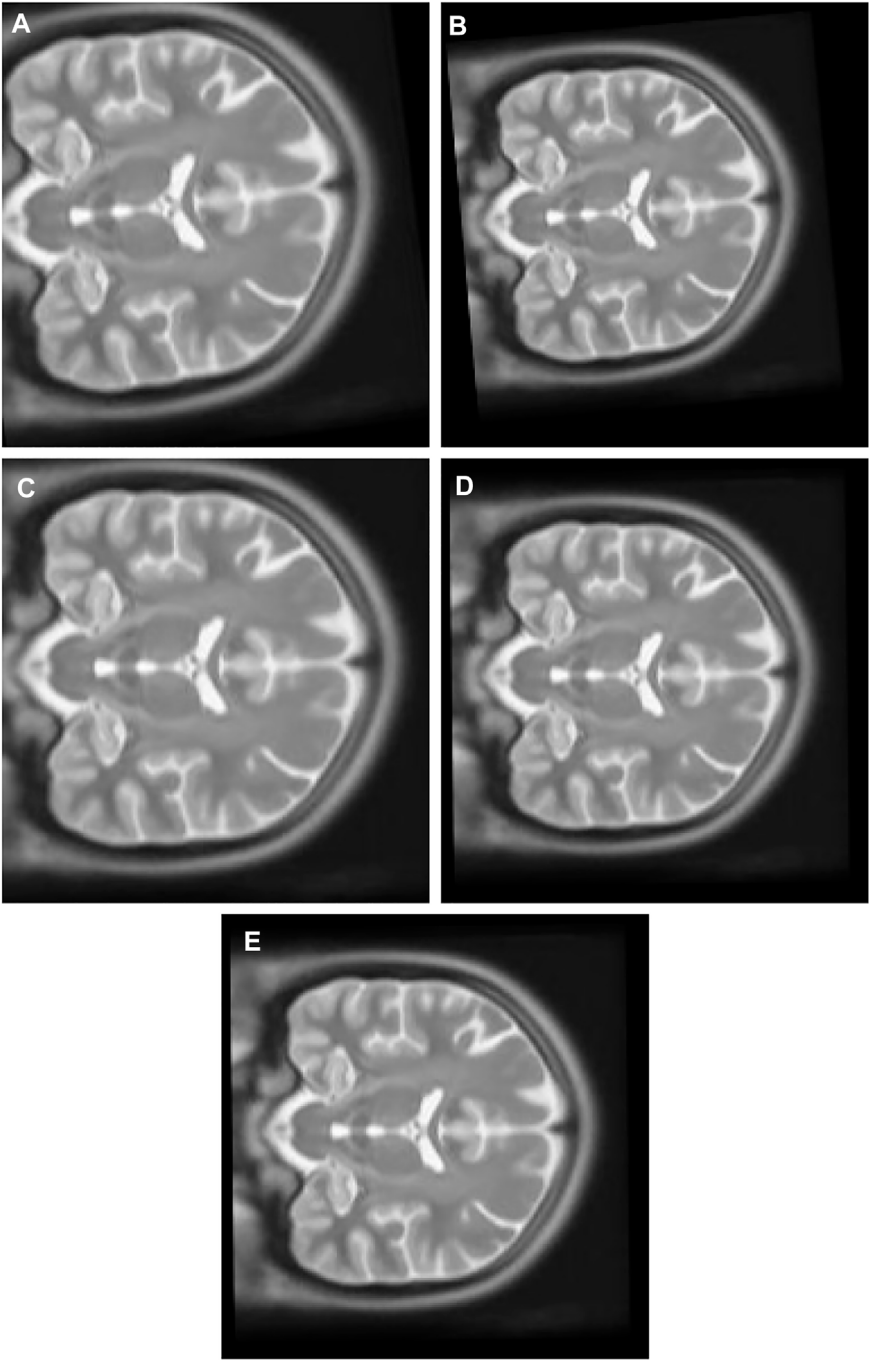
**(A)** Multi Modal Experiment 2 Float: row first left. **(B)** Multi Modal Experiment 2 GA output: row first right. **(C)** Multi Modal Experiment 2 PSO output: row second left. **(D)** Multi Modal Experiment 2 PSO and Powell Output: row second right. **(E)** Multi Modal Experiment 2 TPSysIR Output: row third center.

**FIGURE 11:**
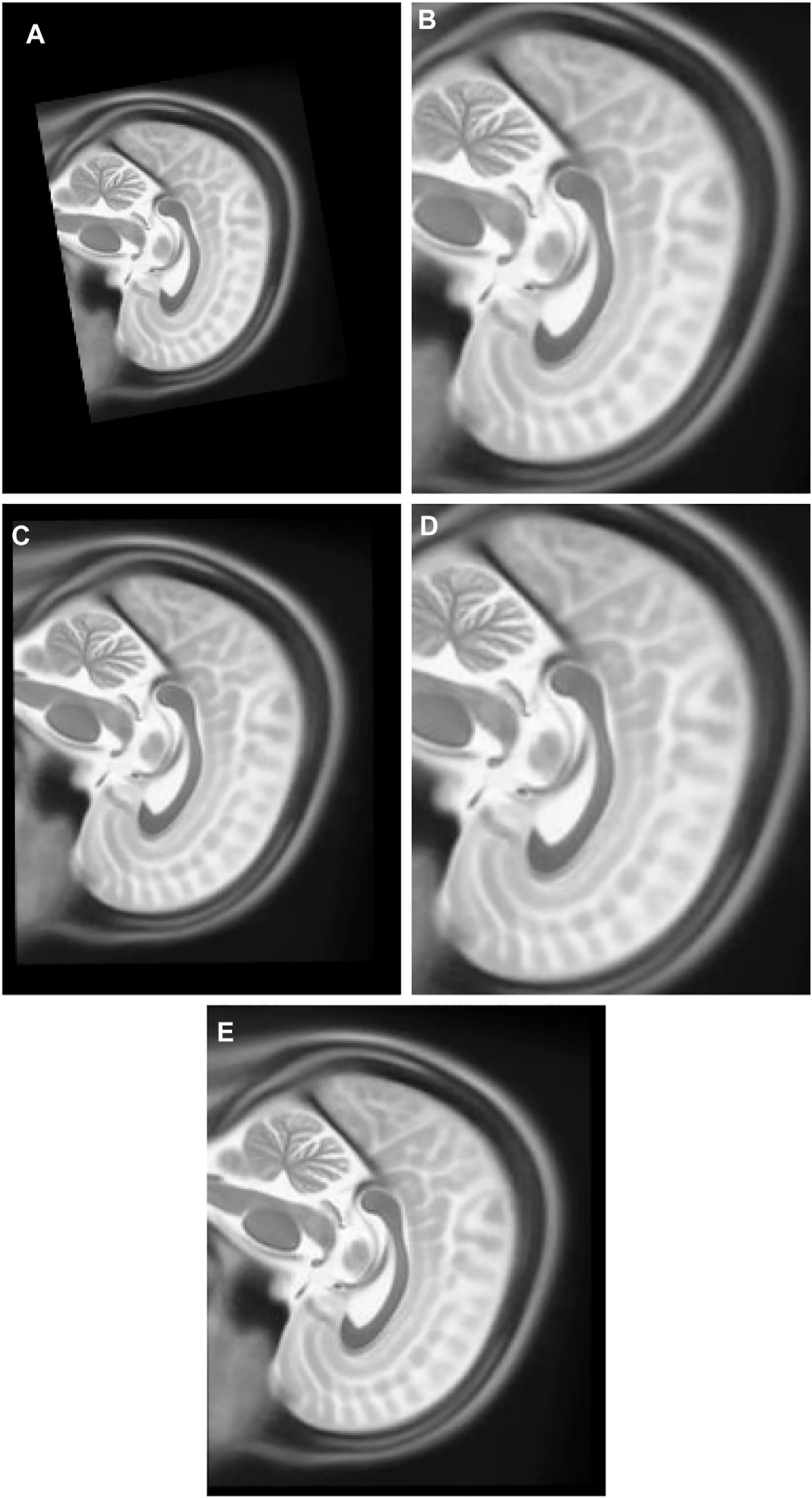
**(A)** Multi Modal Experiment 3 Float: row first left. **(B)** Multi Modal Experiment 3 GA output: row first right. **(C)** Multi Modal Experiment 3 PSO output: row second left. **(D)** Multi Modal Experiment 4 PSO and Powell Output: row second right. **(E)** Multi Modal Experiment 5 TPSysIR Output: row third center.

1) Experiment 1: The multimodal image set one contains images from two different modes, cerebrospinal fluid (CSF) section T1-weighted MRI Figure 8B having low signal and T2-weighted Figure 8A having high signal in the CSF section. T1 in X-Y plane image was utilized to create the float image Figure 9A after it was panned eight pixel units in *x* axis in the upward direction, six pixel units in *Y* axis in the left direction, rotated 5° in a counter clockwise direction, and scaled into 0.8 of its original size.

2) Experiment 2: The multimodal image set two contains T1 Figure 8C and T2 Figure 8D in the X-Z plane. The float Figure 10A was created from T2 after it was panned 10 pixel units in *X* axis in the upward direction, 13 pixel units in *Z* axis in the left direction, rotated 7° in a counter clockwise direction, and scaled into 1.1 of its original size. T1 is the target image.

3) Experiment 3: The multimodal image set two contains T1 and T2 images in the Y-Z plane. T1 Figure 8E is the target image while T2 Figure 8F was utilized as the float image. Figure 11A was created after it was panned 12 pixel units in *Y* axis in the upward direction, nine pixel units in *Z* axis in the left direction, rotated 10° in a counter clockwise direction, and scaled into 0.7 of its original size.

## Experimental results and analysis

The experiments designed above are applied to the proposed TPSysIR algorithm along with three other optimization-based algorithms, GA, PSO, PSO, and POWELL. The experiments were repeated eight times for each method, and the maximum and minimum variance values were recorded and tabulated. MI value was also calculated for the output configuration on recorded data. The results for the variance along the *X*-axis and *Y*-axis and rotation angle(*θ*) are recorded in Table 1 for experiment 1, utilizing single modal images with no scaling factor. Table 2 shows that scaling factor variance S is considered for experiment 2 utilizing single modal image data with a scaling factor. Table 2 with multimodal experiments 1, 2, and 3 utilizes multimodal images for the experiments, and all four variables are considered.

**TABLE 1:** Without scaling DATA.

Algorithm		***X* − 8**	***Y* − 6**	***θ* − 5**	***MI***
GA	Max	0.925	0.888	0.132	1.459
	Min	0.688	0.722	0.030	1.075
	Mean	0.806	0.805	0.081	1.267
	Δ	0.118	0.083	0.051	0.192
PSO	Max	0.679	0.741	0.111	1.232
	Min	0.490	0.607	0.073	0.962
	Mean	0.584	0.674	0.092	1.097
	Δ	0.094	0.067	0.019	0.135
PSO and POWELL	Max	0.530	0.496	0.072	1.569
	Min	0.493	0.465	0.055	1.356
	Mean	0.511	0.480	0.063	1.462
	Δ	0.018	0.015	0.008	0.106
TPSysIR	Max	0.511	0.508	0.058	1.712
	Min	0.470	0.484	0.036	1.669
	Mean	0.49	0.496	0.047	1.690
	Δ	0.020	0.012	0.011	0.021

**TABLE 2:** With scaling DATA.

**Algorithm**		***X* − 8**	***Y* − 6**	***θ* − 5**	***S-1.25***	***MI***
Single multimodal experiment 2						
GA	Max	0.717	0.678	0.316	0.010	0.385
	Min	0.516	0.123	0.139	0.003	0.291
	Mean	0.616	0.400	0.227	0.006	0.324
	Δ	0.10	0.24	0.08	0.003	0.033
PSO	Max	0.845	0.507	0.234	0.006	0.445
	Min	0.633	0.039	0.132	0.002	0.402
	Mean	0.739	0.273	0.183	0.004	0.423
	Δ	0.106	0.234	0.051	0.002	0.021
PSO and POWELL	Max	0.567	0.461	0.189	0.005	0.519
	Min	0.421	0.055	0.143	0.003	0.477
	Mean	0.494	0.251	0.166	0.004	0.498
	Δ	0.073	0.196	0.023	0.001	0.021
TPSysIR	Max	0.459	0.362	0.189	0.004	0 0.551
	Min	0.400	0.049	0.123	0.002	0.532
	Mean	0.429	0.206	0.156	0.003	0.541
	Δ	0.029	0.156	0.033	0.001	0.019
Multimodal expreiment 1						
GA	Max	0.98	0.48	0.50	0.025	0.0079
	Min	0.75	0.12	0.32	0.018	0.0013
	Mean	0.86	0.30	0.41	0.006	0.0046
	Δ	0.12	0.18	0.09	0.003	0.0033
PSO	Max	0.84	0.31	0.47	0.023	0.0100
	Min	0.62	0.03	0.16	0.019	0.0031
	Mean	0.73	0.17	0.31	0.004	0.0065
	Δ	0.11	0.14	0.11	0.002	0.0034
PSO and POWELL	Max	0.56	0.24	0.30	0.013	0.0112
	Min	0.42	0.05	0.18	0.011	0.0070
	Mean	0.49	0.14	0.24	0.004	0.0091
	Δ	0.07	0.10	0.06	0.001	0.0021
TPSysIR	Max	0.38	0.22	0.25	0.010	0.0136
	Min	0.31	0.03	0.21	0.008	0.0098
	Mean	0.35	0.13	0.23	0.003	0.0112
	Δ	0.03	0.09	0.02	0.001	0.0019
Multimodal experiment 2						
GA	Max	1.655	1.751	0.965	0.074	0.0191
	Min	1.295	1.591	0.074	0.018	0.0071
	Mean	1.475	1.671	0.854	0.062	0.0131
	Δ	0.180	0.80	0.111	0.012	0.0060
PSO	Max	1.126	1.506	0.751	0.060	0.0204
	Min	0.862	1.400	0.419	0.043	0.0115
	Mean	0.994	1.453	0.585	0.051	0.0160
	Δ	0.132	0.053	0.83	0.009	0.0045
PSO and POWELL	Max	0.850	1.118	0.452	0.023	0.0225
	Min	0.664	1.047	0.353	0.015	0.0147
	Mean	0.757	1.082	0.402	0.019	0.0186
	Δ	0.093	0.036	0.050	0.004	0.0039
TPSysIR	Max	0.503	0.643	0.324	0.012	0.0271
	Min	0.381	0.547	0.280	0.010	0.0221
	Mean	0.442	0.608	0.242	0.008	0.0246
	Δ	0.061	0.035	0.040	0.002	0.0024
Multimodal experiment 3						
GA	Max	1.122	1.990	0.965	0.617	0.0308
	Min	0.810	1.796	0.743	0.578	0.0071
	Mean	0.966	1.893	1.204	0.597	0.0235
	Δ	0.156	0.097	0.093	0.020	0.0162
PSO	Max	1.009	1.838	1.067	0.471	0.0387
	Min	0.739	1.445	0.982	0.424	0.0261
	Mean	0.874	1.641	0.984	0.447	0.0324
	Δ	0.135	0.197	0.083	0.023	0.0063
PSO and POWELL	Max	0.598	0.523	0.708	0.163	0.0447
	Min	0.400	0.377	0.568	0.129	0.0147
	Mean	0.499	0.450	0.638	0.146	0.0401
	Δ	0.099	0.073	0.070	0.017	0.0355
TPSysIR	Max	0.323	0.361	0.531	0.088	0.0544
	Min	0.187	0.267	0.460	0.062	0.0476
	Mean	0.237	0.314	0.495	0.075	0.0510
	Δ	0.086	0.047	0.035	0.013	0.0034

**FIGURE 12:**
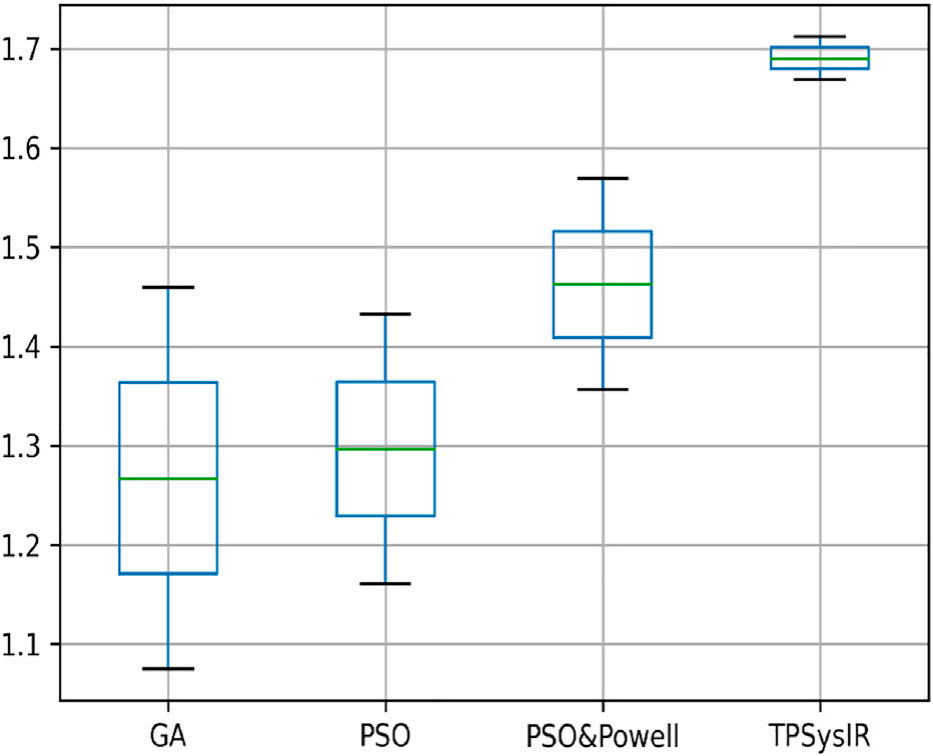
Mutual Information ranges in each algorithm for Table 1 single modal experiment 1.

Table 1 results with no size scaling experiment show that PSO and POWELL have the lowest mean variance (Mean) and deviation(Δ) among the results in the *Y*-axis. TPSysIR has the lowest variance value on the *X*-axis and equals the deviation to PSO and POWELL. The mean rotation variance (*θ*) is the least in TPSysIR than all other algorithms including GA, PSO, and PSO and POWELL. TPSysIR has the maximum values, in mutual information, of 1.76 and the slightest deviation 0.02. The boxplot Figure 12 shows that the range of MI values obtained by PSO and Powell and TPSysIR is better than other algorithms, but TPSysIR obtains the range of least deviation. The maximum MI results for TPSysIR has shown 17.93%, 19.58%, and 9.61% improvement against the corresponding maximum MI values of GA, PSO, and PSO and POWELL algorithms.

**FIGURE 13:**
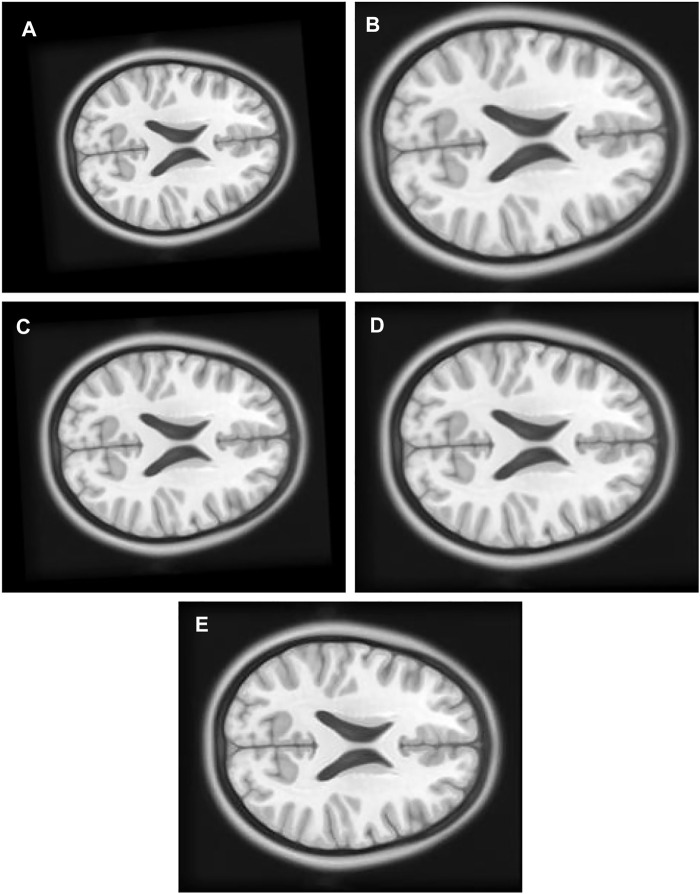
**(A)** Single Modal Experiment 2 Float: row first left. **(B)** Single Modal Experiment 2 GA output: row first right. **(C)** Single Modal Experiment 2 PSO output: row second left. **(D)** Single Modal Experiment 2 PSO and Powell Output: row second right. **(E)** Single Modal Experiment 2 TPSysIR Output: row third center.

**FIGURE 14:**
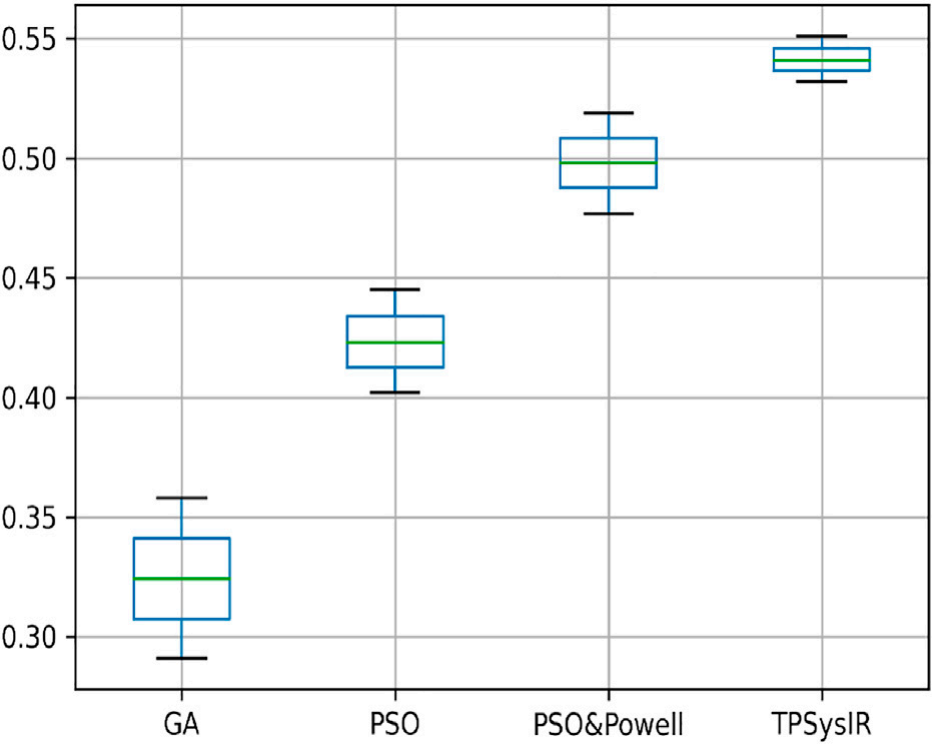
Mutual Information ranges in each algorithm for Table 2 single model experiment 2.

Table 2 single modal experiment 2 data includes the scaling factor along with all other parameters measured in experiment 1 to obtain results from all four methods. Notably in this experiment 2, the overall MI is lower than the previous experiment 1 as the similarity between the original and output image has decreased. The deviation in the *X*-axis is least in the TPSysIR algorithm. The deviation angle of rotation varies from 0.08 to 0.02 for GA to PSO and POWELL and TPSysIR. TPSysIR has a deviation value of 0.03 but has a lower max value than PSO and POWELL. The results of the experiments are shown in Figure 13B–E. PSO and POWELL and TPSysIR did a better job on image scaling. Mutual interference values show slightly better results for TPSysIR than PSO and POWELL, whereas other algorithms such as GA and PSO have lower scores. Angular bias is present in the output configuration images for GA Figure 13B and PSO Figure 13C. The boxplot Figure 14 shows that all algorithms gave minimum deviation in the range of MI values, but the maximum value range was obtained by TPSysIR to obtain best alignment. The maximum MI results for TPSysIR have shown 57.14%, 25%, and 7.84% improvement against the corresponding maximum MI values of GA, PSO, and PSO and POWELL algorithms, respectively.

**FIGURE 15:**
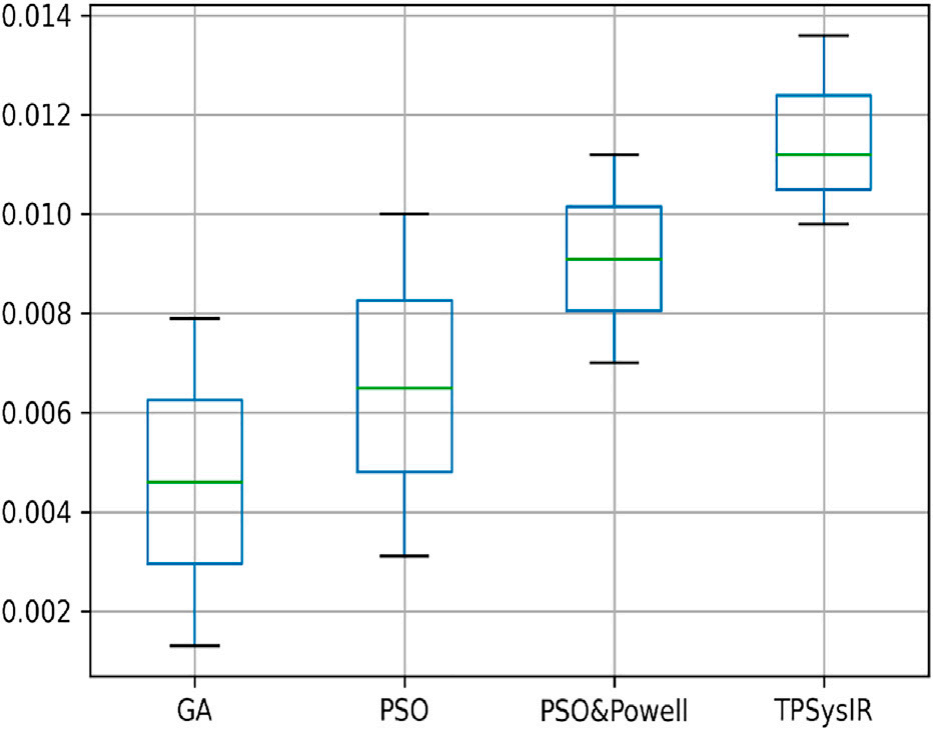
Mutual Information ranges in each algorithm for Table 2 Multimodal Experiment 1.

Table 2 multimodal experiment 1 data shows lower ranges of the MI values than in previous experiments, expressing further dissimilarity between the reference and the output image. The TPSysIR algorithm shows lower deviation values for X, Y, and rotation angle variance. The scalability factor deviation has relative range values for PSO and POWELL and TPSysIR, while MI values are best for TPSysIR. The boxplot Figure 15 shows that the ranges of MI values obtained by TPsysIR were the best. The maximum MI results for TPSysIR has shown 72.15%, 36%, and 21.42% improvement against corresponding maximum MI values of GA, PSO, and PSO and POWELL algorithms, respectively. The configuration results obtained for each method in the experiment for multimodal images are shown in Figure 9B–E.

**FIGURE 16:**
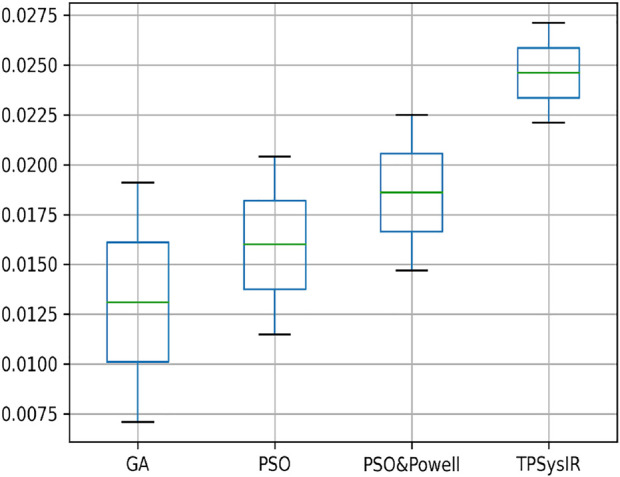
Mutual Information ranges in each algorithm for Table 2 Multimodal Experiment 2.

Table 2 multimodal experiment 2 contains the data from the experiment performed over multimodal T1 and T2 images taken in the X-Z plane. The output in Figure 10B–E shows the output for the experiment. The table values for GA and PSO variance over X and Z have significantly higher values than PSO and Powell and TPSysIR. The MI value shows overlapping ranges for GA, PSO, and PSO and Powell while TPSysIR has higher ranges, as shown in the boxplot Figure 16. The TPSysIR has 29.52%, 24.72%, and 16.97% better MI values than GA, PSO, and PSO and Powell.

**FIGURE 17:**
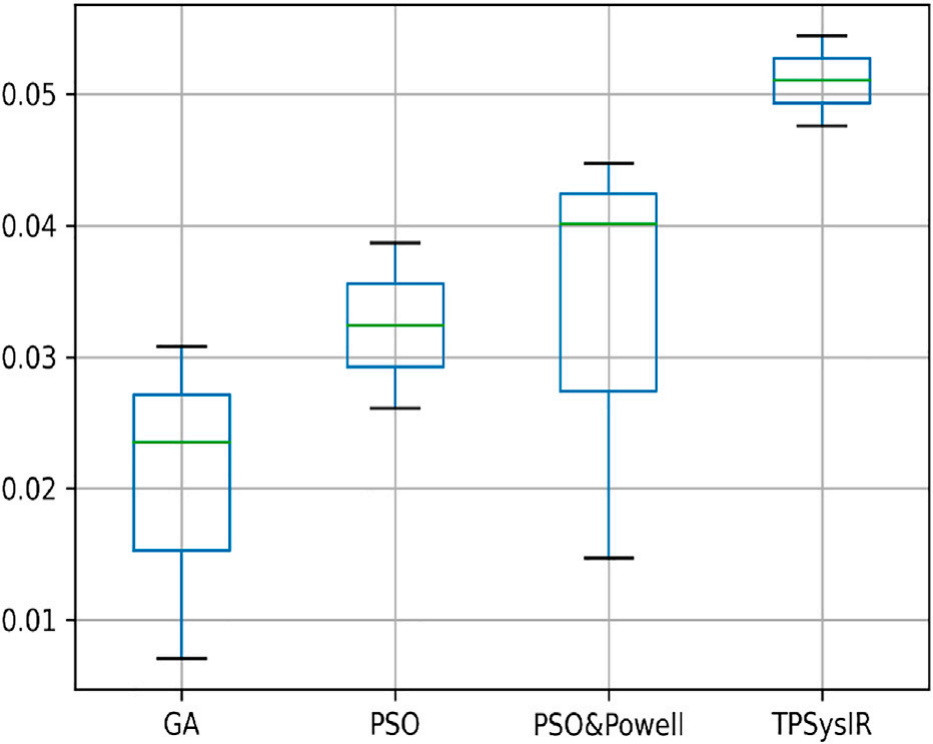
Mutual Information ranges in each algorithm for Table 2 Multimodal Experiment 3.

Table 2 multimodal experiment 3 data is obtained from the experiment performed over multimodal T1 and T2 images taken in the Y-Z plane. The output data for this experiment are shown in Figures 11B–E. The data shows the variance in Y of GA is the maximum of all other algorithms, and TPSysIR has the lowest. The MI range values of GA, PSO, and PSO and Powell show overlapping, while TPSysIR has a higher range of values. The same is confirmed in the boxplot in Figure 17. The TPSysIR MI values are 43.38%, 28.86%, and 17.83% are better than GA, PSO, and PSO and Powell respectively.

PSO and POWELL has good optimization capability due to its hybridization to acquire local and global search capabilities. The TPSysIR algorithm employs the TPS’s parallel execution capability, optimizing the local search and creating a globally optimal solution with faster convergence of results.

## Conclusion

The method described in this paper utilizes the tissue P system’s parallel and simultaneous execution feature to guide its velocity position model-based rules. The novelty of this work is the use of the TPS, which enables faster convergence and high MI with the parallel feature using an optimization-based model to obtain parameters for image registration and the use of the PSO technique to make the evolution rules. The algorithm is tested on multimodal and unimodal MRI image sets to verify its effectiveness. The results of the tests prove it to be a good optimization-based solution to the image registration problem compared to other state-of-the-art algorithms.

## Data Availability

The datasets presented in this study can be found in online repositories. The names of the repository/repositories and accession number(s) can be found at: McConnell Brain Imaging Centre. https://www.bic.mni.mcgill.ca/Ser- vicesAtlases/ICBM152NLin2009.
